# Should we routinely assess hypothalamic–pituitary–adrenal axis in pediatric patients with Prader–Willi syndrome?

**DOI:** 10.3389/fendo.2024.1406931

**Published:** 2024-06-27

**Authors:** Anna Maria Wędrychowicz, Katarzyna Doleżal-Ołtarzewska, Agata Zygmunt-Górska, Anna Urszula Kalicka-Kasperczyk, Katarzyna Tyrawa, Malgorzata Wojcik, Dominika Janus, Adrianna Kot, Agnieszka Lecka-Ambroziak, Elzbieta Petriczko, Joanna Wielopolska, Jerzy Starzyk

**Affiliations:** ^1^ Department of Pediatric and Adolescent Endocrinology, Jagiellonian University Medical College, Kraków, Lesser Poland, Poland; ^2^ Department of Pediatric and Adolescent Endocrinology, University Children’s Hospital of Cracow, Cracow, Poland; ^3^ Department of Endocrinology and Diabetology, The Children’s Memorial Health Institute, Warsaw, Masovian, Poland; ^4^ Endocrinology Outpatient Clinic, Institute of Mother and Child, Warsaw, Masovian, Poland; ^5^ Department of Pediatrics, Endocrinology, Diabetes, Metabolic Disorders and Cardiology of Developmental Age, Pomeranian Medical University, Szczecin, West Pomeranian, Poland

**Keywords:** Prader-Willi syndrome (PWS), hypothalamic-pituitary-adrenal axis (HPAA), central adrenal insufficiency (CAI), low-dose ACTH test (LDAT), glugacon stimulation test (GST)

## Abstract

**Background:**

It has been reported that central adrenal insufficiency (CAI) in pediatric patients (pts) with Prader–Willi syndrome (PWS) may be a potential cause of their sudden death. In addition, the risk of CAI may increase during treatment with recombinant human growth hormone (rhGH).

**Objective:**

To prevent both over- and undertreatment with hydrocortisone, we evaluated the prevalence of CAI in a large multicenter cohort of pediatric pts with PWS analyzing adrenal response in the low-dose ACTH test (LDAT) and/or the glucagon stimulation test (GST) and reviewing the literature.

**Methods:**

A total of 46 pts with PWS were enrolled to the study, including 34 treated with rhGH with a median dose of 0.21 mg/kg/week. LDAT was performed in 46 pts, and GST was carried out in 13 pts. Both tests were conducted in 11 pts. The tests began at 8:00 a.m. Hormones were measured by radioimmunoassays. Serum cortisol response >181.2 ng/mL (500 nmol/L) in LDAT and >199.3 ng/mL (550 nmol/L) in GST was considered a normal response. Additionally, cortisol response delta (the difference between baseline and baseline) >90 ng/mL and doubling/tripling of baseline cortisol were considered indicators of normal adrenal reserve.

**Results:**

Three GSTs were not diagnostic (no hypoglycemia obtained). LDAT results suggested CAI in four pts, but in two out of four pts, and CAI was excluded in GST. GST results suggested CAI in only one patient, but it was excluded in LDAT. Therefore, CAI was diagnosed in 2/46 pts (4.3%), 1 treated and 1 untreated with rhGH, with the highest cortisol values of 162 and 175 ng/dL, but only in one test. However, in one of them, the cortisol delta response was >90 ng/mL and peak cortisol was more than tripled from baseline. Finally, CAI was diagnosed in one patient treated with rhGH (2.2%).

**Conclusion:**

We present low prevalence of CAI in pediatric pts with PWS according to the latest literature. Therefore, we do not recommend to routinely screen the function of the hypothalamic–pituitary–adrenal axis (HPAA) in all pts with PWS, both treated and untreated with rhGH. According to a review of the literature, signs and symptoms or low morning ACTH levels suggestive of CAI require urgent and appropriate diagnosis of HPAA by stimulation test. Our data indicate that the diagnosis of CAI should be confirmed by at least two tests to prevent overtreatment with hydrocortisone.

Our study presents the low prevalence of central adrenal insufficiency (CAI) in a multicenter cohort of pediatric patients with Prader–Willi syndrome (PWS) and summarizes 15 years of research on this issue. A detailed analysis of the results allows us to unequivocally exclude the need for routine diagnostics for CAI in this group of patients. The results of our work indicate that none of the diagnostic tests are sensitive enough to detect CAI, so the suspicion of CAI based on the result of one test should be confirmed in another. The prevalence of CAI is low in PWS and does not appear to increase with the introduction of growth hormone therapy. The diagnosis should be performed in patients with signs and symptoms suggestive of CAI and low morning ACTH levels.

## Introduction

Prader–Willi syndrome (PWS) is a recognizable pattern of physical impairment with significant cognitive, neurological, endocrine, and behavioral abnormalities caused by a lack of gene expression from the impaired region inherited from the father of chromosome 15q11-q13, near the centromere (1). Patients with PWS develop hypothalamic dysfunction that can lead to various endocrine disorders (2). Besides the well-known endocrine abnormalities such as GH deficiency, hypogonadism, or pathological obesity, some studies over the last 15 years revealed the possibility of central adrenal insufficiency (CAI), the prevalence of which has been variously described in pediatric patients with PWS. These clinical observations were inspired by a study from 2008 by de Lind van Wijngaarden et al., which found that 60% of patients with PWS present CAI diagnosed on the basis of the test with metyrapone (3). Subsequent studies in 2010 showed a significantly lower prevalence of CAI (4%) based on the ACTH test and the insulin test (4) or did not confirm the presence of CAI in pediatric patients with PWS based on the ACTH test (5). Further studies also did not confirm such a high prevalence of CAI in patients with PWS (6–8). In the study by Corrias et al., CAI was confirmed in 14.3% of patients with PWS based on the low-dose ACTH test (LDAT) and in 4.8% of patients with PWS based on the standard-dose ACTH test (SDAT) (6). The latest studies in pediatric populations excluded CAI in patients with PWS based on the test with insulin (7) and based on the test with metyrapone, but not LDAT (8). Thus, the question “can CAI be part of the PWS phenotype in children?” still remains unanswered.

In addition, it is hypothesized that the risk of CAI may increase during rhGH treatment and may be a potential cause of sudden death in patients with PWS. GH inhibits 11β-hydroxysteroid dehydrogenase type 1 (11β-HSD1), resulting in decreased conversion of cortisone to active cortisol. Therefore, it was postulated that patients with PWS treated with rhGH should be monitored for the possibility of an episode of adrenal insufficiency (2). The next question that needs to be answered is: Should the hypothalamic–pituitary–adrenal axis (HPAA) be routinely evaluated in all patients with PWS treated with rhGH? There is also no agreement on the prevalence of CAI in adults with PWS (9, 10). In some countries, it has been common practice to prescribe stress-dose hydrocortisone during physical or psychological stress in patients with PWS (6, 11). The side effects of frequent use of hydrocortisone include weight gain, osteoporosis, diabetes, and hypertension—already serious problems in adults with PWS. However, inadequate treatment of CAI can cause significant morbidity and even mortality. The most recent data from 2020 indicate that CAI is rare (1.2%) in adults with PWS (12). Based on multicenter data obtained from 82 patients, the authors advise against routine prescribing stress doses of hydrocortisone in adults with PWS. Therefore, it is time to ask again, what are the accurate and true data on CAI in children with PWS?

To prevent both over- and undertreatment with hydrocortisone in children with PWS, we evaluated adrenal response in LDAT and/or GST in a large multicenter cohort of pediatric patients with PWS. The aim of the study was to assess HPAA in patients with PWS (1) to obtain information about the prevalence of CAI in patients with PWS (2), to compare the results of HPAA diagnostic tests (3), to compare the results obtained in patients with PWS treated and untreated with rhGH, and (4) to analyze the results with the currently available literature in order to propose common recommendations for the assessment of HPAA in patients with PWS.

## Material and methods

### Patients

We conducted a prospective cohort study. A total of 46 consecutive patients with PWS were enrolled in the study between 2014 and 2023, first in Krakow, and then, from 2021, in Warsaw and Szczecin. Thirty-four of them (74%) were treated with rhGH, and 12 were managed without rhGH treatment (before its introduction or because of severe obesity). We did not plan a control group. The study was approved by the Ethics Committee of the Jagiellonian University (consent number: KBET/212/B/2012 of 28 June 2012 and its updates of 4 April 2023, 27 September 2017, and 23 January 2020). All participants over the age of 16 and their parents have given written informed consent. Inclusion criteria were genetic confirmation of PWS by genetic testing, and the exclusion criteria were as follows: lack of parental consent to the study and acute infection at the time of the study (in these cases, the tests were postponed). In patients on steroid therapy, including inhaled steroid therapy, tests were performed after discontinuation of steroids after the time dependent on the steroid used. The clinical characteristics of patients with PWS are presented in **Table 1**.

**Table 1:** Clinical characteristics of patients with Prader–Willi syndrome.

Pts with PWS	N	Female	Male	Mean age (years)	Mean bone age (years)	Mean height (SD)	Mean BMI (SD)	Mean rhGH dose (mg/kg/week)	Median IGF-1 (SD)	HbA1c (%)
Treated with rhGH	34	16	18	9.5	9.7	−0.30	2.30	0.21	1.8	5.3
Untreated with rhGH	12	6	6	11.7	11.9	−2.84	2.58	–	−1.6	5.4

### Methods

After an initial medical history and physical examination, HPAA assessment was performed on all enrolled patients using LDAT and/or GST. The aim of the project was to perform both tests in all patients in order to diagnose HPAA and compare the tests. It was not possible to achieve this assumption in all patients due to the temporary lack of synthetic ACTH or the lack of consent of the patients’ parents for another test, when the first one ruled out adrenal insufficiency.

### Low-dose ACTH test

The patient was installed with a venous sampling catheter early in the morning around 7:00–7:30. At 8:00 a.m., synthetic ACTH (tetracosactide) was administered intravenously at an absolute dose of 1 μg or 0.5 μg/m^2^ of body surface area. Serum cortisol and plasma ACTH were measured at baseline, then cortisol levels were measured 20, 30, and 60 min after ACTH administration.

### Glucagon stimulation test

The installation of the catheter for venous sample collection was done in the same way as in LDAT. Around 8:00 a.m., glucagon was administered intramuscularly at a dose of 0.1 mg/kg (maximum dose of 1.0 mg) (12). Plasma ACTH and serum cortisol were measured at baseline. Blood glucose levels were measured at baseline and every 30 min up to 180 min after glucagon injection. In addition, blood for serum cortisol measurement was drawn every 30 min from 90 min to 180 min after glucagon injection, at the time of expected onset of hypoglycemia.

### Normal ranges and interpretation of tests

Serum cortisol response >181.2 ng/mL (500 nmol/L) in LDAT and >199.3 ng/mL (550 nmol/L) in GST was considered a normal response. In addition, the cortisol response delta (the difference between baseline and its highest value) >90 ng/mL or doubling/tripling of baseline cortisol indicates normal adrenal reserve. The normal range for morning ACTH is 10–60 pg/mL, and that for morning cortisol is 50–230 ng/mL (13–17).

### Biochemical methods

Plasma ACTH and serum cortisol levels were measured by radioimmunoassays according to the instructions of their producers (ACTH—Brahms, Germany and cortisol—Siemens Healthcare Diagnostics, USA).

### Statistical analysis

The results were statistically analyzed using the Dell Statistica 13.1 64-bit package (StatSoft, Poland, Kraków). Results are presented as a median (95% confidence interval) or as a mean ± SD or as %. To test if the variance in cortisol levels was different between the two groups (patients with PWS treated and untreated with rhGH) and between the two stimulation tests (ITT and GT), analysis of variance was used. A *p*-value of less than 0.05 was considered statistically significant.

## Results

We conducted a total of 58 tests: 45 LDATs and 13 GSTs. Both tests were performed on 11 patients. We did not observe any serious side effects from either test. Three GSTs were not diagnostic (no hypoglycemia was achieved). Two patients had LDATs twice, due to repeated low cortisol levels in the morning without a clinical CAI picture.

LDAT results suggested CAI in four patients, but in two out of four patients, CAI was excluded at GST. GST suggested CAI in only one patient, but was excluded in LDAT. Therefore, CAI was diagnosed in 2/46 patients (4.3%), 1 treated and 1 untreated with rhGH, with the highest cortisol values of 162 and 175 ng/dL, but only on the basis of one test. However, in one of them, the cortisol delta response was >90 ng/mL and more than tripled from baseline. Finally, CAI was diagnosed in one PWS patient treated with rhGH (2.2%), but only on the basis of LDAT. The results of the diagnostic tests are presented in **Table 2**.

**Table 2:** Results of diagnostic tests for CAI in patients with PWS. Median and ranges are presented. LDAT, low-dose ACTH test; GST, glucagon stimulation test.

Tests	Basal ACTH (pg/mL), normal range, 10–60 pg/mL	Basal cortisol (ng/mL), normal range, 50–230 ng/mL	Peak cortisol (ng/mL)	Delta of cortisol in test (ng/mL)	No. of tests with doubling/triplication of baseline cortisol
**LDAT in treated with rhGH**	10.2–61.3 28.1	34.6–190.4 93.5	162–355.8 245.6	64.3–287.3 152.4	30/33
**LDAT in untreated with rhGH**	22.2–24.9 23.5	43.5–159.7 82.5	165.4–316.8 223.3	75–187.9 117.7	8/10
**GST in treated with rhGH**	10.2–45.5 35	55.3–179.4 73.9	161.2–312.5 246.6	93.6–246.1 175.8	7/8
**GST in untreated with rhGH**	15.2–37.3 26.8	54–73.4 63.7	233.9–245.3 239.2	179.9–209.8 194.9	2/2
**LDAT**	10.2–61.3 27.5	34.6–190.4 79.0	162–355.8 235.2	64.3–287.3 152.2	38/43
**GST**	10.2–45.5 29.5	54–179.4 80.4	161.2–312.5 236.0	93.6–246.1 179.9	9/10

Morning/basal cortisol levels were within the normal range in all patients with PWS not treated with rhGH. Low morning/basal cortisol levels were observed in 4 of the 34 patients treated with rhGH (12%); however, in all of these cases, CAI was excluded by dynamic testing. In contrast, the only CAI patient diagnosed only by LDAT had normal morning cortisol. All patients, both treated and untreated with rhGH, had normal morning ACTH levels. None of our patients developed signs and symptoms of adrenal insufficiency, and none of them were treated with hydrocortisone on a permanent basis. In our only patient with CAI, we recommend treatment in a stressful situation, and we plan to perform another HPAA assessment test.

We found no differences in morning ACTH and cortisol values, peak cortisol and cortisol delta between LDAT and GST, and between patients with PWS treated and untreated with rhGH.

## Discussion

The results of our study confirmed a very low prevalence of CAI in patients with PWS, consistent with most previous references (**Table 3**). Data reports previously from different clinical centers differed even when the same diagnostic tests were used to assess HPAA. The key issue seems to be the correct diagnosis of CAI in this group of patients. It is well known that CAI cannot be determined in patients with PWS by measuring cortisol levels under stressful conditions such as high fever, as patients with PWS may not present with fever during severe infection due to hypothalamic disorders. Therefore, a stimulation test may be the best way to detect adrenal insufficiency. There are several tests used for CAI detection: LDAT, metyrapone test, insulin-induced hypoglycemia test (IIHT), glucagon stimulation test (GST), and corticotrophin hormone (CRH) stimulation test (13, 14). Which test is the best? The IIHT is the gold standard for evaluating the presence of CAI, but it can be dangerous especially in patients with PWS due to the possibility of decreased count regulation hormone response. Metyrapone testing can induce an acute adrenal crisis in patients with a chronic glucocorticoid deficiency and induces sensations such as nausea, vomiting, and hypotension—symptoms of cortisol deficiency also in healthy individuals. The results of the CRH stimulation test, which evaluates the ability of the pituitary gland to secrete ACTH to stimulate cortisol production, are difficult to interpret. There are conflicting reports regarding the cutoff points for this test, and furthermore, the availability of CRH is problematic in some countries. LDAT has high sensitivity in the diagnosis of adrenal dysfunction, but may miss benign CAI (15, 16). The GST has the same sensitivity as LDAT for diagnosing CAI, but may pick up more subtle abnormalities of the HPAA. It is considered an equal and safe alternative to the IIHT and produces similar cortisol responses. An obtained hypoglycemia in both tests is a fast and potent stimulus of CRH secretion, which will, in turn, enhance ACTH secretion and thereafter cortisol production. Thus, a normal response to hypoglycemia requires integrity of the entire HPAA (14).

**Table 3:** Results of studies assessing the prevalence of central adrenal insufficiency in pediatric patients with Willi–Prader syndrome.

Study	*N*	Median age (range)/mean age (SD)*	Testing method	Cutoff points in tests	Treated with GH/untreated (%)	Prevalence of CAH (%)
**de Lind van Wijngaarde et al. (3)**	25	9.7 (3.7–18.6)	MT	11-DOC >200 nmol/L (7 µg/dL)	25/0 (100%)	**60**
**Connell et al. (4)**	4 6 15	7.16 (0.43–16.27)	LDAT SDAT IIHT	Cortisol >18 μg/dL (500 nmol/L) for all tests	Lack of data	**4** **(1 pt in IIHT)**
**Nyunt et al. (5)**	41	7.68 (± 5.23)*	LDAT	Cortisol >18 μg/dL (500 nmol/L) for all tests	19/22 (46%)	**0**
**Corrias et al. (6)**	84 (9 had both tests)	7.7 (± 5.0)*	LDAT HDST	Cortisol >18 μg/dL (500 nmol/L)	53/31 (63%)	**14.2** **4.8**
**Beauloye et al. (15)**	20: 14 7 (1 had both test)	4.55 (0.8–14.7) 5.6 (3.5–14.4)	GST IIHT	Cortisol >20 μg/dL (550 nmol/L) and/or delta cortisol* >9.0 μg/dL (250 nmol/L) for both tests	5/15 (25%)	5/0*
**Obrynba et al. (8)**	21	13.9 (± 10.9)*	LDAT MT	Cortisol ≥15.5 μg/dL (428 nmol/L) 11-DOC >200 nmol/L (7 µg/dL)	16/5 (76%)	**29** **0**
**Oto et al. (7)**	36	2.0 (0.6–12.0)	IIHT	Cortisol >18 μg/dL (500 nmol/L)	0/36 (0%)	**0**
**Angulo et al. (18)**	128	8.63 (± 5.3)* (0.3–18)	No test, only morning ACTH and cortisol	Normal ranges: Cortisol 3–25 μg/dL (83–690 nmol/L) ACTH 9–57 pg/mL (2–13 pmol/L)	128/0 (100%)	**0**
**Grootjen et al. (19)**	93 (30 had two times) (11 had three times) (1 had four times)	0.97 (0.48–4.81)	MT	ACTH peak >13 pmol/L (59 pg/mL) 11-DOC >200 nmol/L (7 µg/dL)	75/18 (76%) 30/0 (100%) 11/0 (100%)	**0**
**Our study**	46: 45 (2 patients had two times) 13 (11 had both tests)		LDAT GST	>181.2 ng/mL (500 nmol/L) >199.3 ng/mL (550 nmol/L) and/or delta cortisol >9.0 μg/dL (250 nmol/L) or doubling/triplication of baseline cortisol for both tests	34/46 (74%)	**2.2**

CAI, central adrenal insufficiency; MT, metyrapone test; LDAT, low-dose ACTH test; HDAT, high-dose ACTH test; IIHT, insulin-induced hypoglycemia test; GST, glucagon stimulation test; delta cortisol—an increase of cortisol calculated between the lowest cortisol and the highest cortisol level during the stimulation test.

*-mean age (SD).

The highest incidence of CAI was reported by the Dutch team in 2018 (3). The authors performed only one test with metyrapone in the diagnosis of CAI. All of their patients underwent this examination during an overnight stay in the Pediatric Intensive Care Unit. Further studies have shown a significantly lower incidence of CAI or even its absence in patients with PWS (4–8). Recent data indicate that CAI is not a crucial clinical problem in PWS, but these patients do have a delayed HPAA response during acute stress (7). In the study by Oto et al., basal and peak cortisol levels were within the normal range, but the peak cortisol response to ITT was delayed in the majority of patients with PWS (64%) (7). Although the mechanism remains unclear, this delay may indicate the existence of a central obstacle to HPAA adjustment. This delayed HPAA response during acute stress has also been reported in a recent study by Grootjen et al. (19). Perhaps in the study by Wijngarden et al., delayed adrenal response was not included in the final results (3). In contrast, in the study by Obrynba et al., CAI was excluded in all 21 participants with PWS using the metyrapone test, although 29% of them simultaneously failed LDAT (8). Analyzing the results of this study, it is worth noting that the authors started LDATs in all patients at 10:00 p.m. This is the period of the day when cortisol levels are at their lowest, which is why such a high incidence of CAI can be a false-negative test result. Moreover, after the LDAT was completed, metyrapone was administered at midnight on the same day. The adrenal glands were “primed” with ACTH, and this may be the reason why the cortisol response in this test with metyrapone was not delayed (8). Grootjen et al., who also used the test with metyrapone, excluded CAI in all patients with PWS (19). However, in almost 30% of their patients, the authors repeated tests. In some patients, they even repeated the test more the twice. In all studies that used the metyrapone test, the majority of patients were treated with rhGH. Overall, two of the three studies diagnosing HPAA in patients with PWS using the test with metyrapone excluded CAI, and one indicated a high incidence of CAI. LDAT was used to diagnose HPAA in patients with PWS in five studies. In two of them, it allowed the exclusion of CAI (4, 5). In one analyzed previously, the incidence of CAI was 29% but most likely due to false-positive results (8). In the study by Corrias et al., LDAT indicated a CAI prevalence of 14.2%, but it was verified with the standard ACTH test and the prevalence dropped to 4.8% (6). However, there are opinions that the standard ACTH test is not a good tool in the diagnostics of CAI (14), and there is a report indicating that both the standard test and LDAT had a similar diagnostic accuracy in adults and children using different peak serum cortisol cutoff values (16). In our study, LDAT allowed for the diagnosis of CAI in one patient (2.2% of all patients with PWS studied), but it was not confirmed by another test. HIIT was used in three studies, and two of them excluded CAH in all patients with PWS (7, 17), while one study reported a low 6% prevalence of CAI but was not verified by another test (4). In a study by Beauloye et al., CAI was suspected based on an insufficient peak cortisol value of 16.6 μg/dL after insulin injection, but this patient’s cortisol delta was 9.97 μg/dL and CAI was eventually ruled out (15). Two studies used GST in the diagnostics of HPAA, and in both studies, CAI was excluded (17).

Untreated GH deficiency may mask CAI. Low insulin-like growth factor 1 (IGF-1) levels result in increased expression and activity of 11β-HSD1, the enzyme that converts cortisone to cortisol (20). Our study, or none of the previously cited studies, is unlikely to confirm a strong association between CAI prevalence and rhGH treatment. Studies assessing HPAA in patients with PWS untreated with rhGH or treated in the minority did not report cases with CAI (5, 7, 17). When patients were treated with rhGH, the prevalence of CAH amounted to 0%–60%; however, only in one study was this prevalence high—60% (3); in was low in two studies: 2.2% (our data) and 4.8% (6), and three studies found no evidence of CAI (8, 18, 19).

Based on our data and a review of the literature on the incidence of CAI in children and adolescents with PWS, we do not recommend a routine diagnostics of HPAA in this group of patients. If the clinical picture suggests CAI in patients with PWS, an appropriate test should be used and confirmed with another test. Our study, similar to that of Obrynda et al. and Grootjen et al., clearly indicates that the suspicion of CAI in one test should be verified by another test (8, 19). It is known that cortisol levels in a stressful situation are not a good diagnostic tool in the diagnosis of HPAA in patients with PWS. However, based on the results of recent data published by Angulo et al., morning ACTH and cortisol levels may be reliable and helpful in monitoring HPAA function (18). The authors found that patients with PWS had significantly lower morning cortisol levels than the control group, although in both groups, morning cortisol and ACTH levels were in the normal range. Our study did not confirm this observation because in 12% of our patients with PWS treated with rhGH, morning cortisol levels were below normal, but tests ruled out CAI in all of them. In contrast, the only patient with CAI confirmation in single LDAT had normal morning cortisol levels. Since all of our patients with WPS treated and untreated with rhGH had normal morning ACTH levels, it was the morning ACTH level that appears to be more reliable for CAH screening in this group of patients.

## Conclusions

The low incidence of CAI in pediatric patients with PWS is presented according to the latest literature. Therefore, routine screening of HPAA function is not recommended for all patients with PWS, both treated and untreated with rhGH. According to the review of the literature, signs and symptoms or low morning ACTH levels suggestive of CAI require urgent and appropriate diagnosis of HPAA by stimulation test. Our data indicate that the diagnosis of CAI should be confirmed by at least two tests to prevent overtreatment with hydrocortisone.

## Data availability statement

The original contributions presented in the study are included in the article/supplementary material. Further inquiries can be directed to the corresponding author.

## Ethics statement

The studies involving humans were approved by the Ethics Committee of the Jagiellonian University (consent number: KBET/212/B/2012 of 28.06.2012 and its updates of 4.04.2023, 27.09.2017 and 23.01.2020). The studies were conducted in accordance with the local legislation and institutional requirements. Written informed consent for participation in this study was provided by the participants’ legal guardians/next of kin.

## Author contributions

AW: Conceptualization, Data curation, Formal analysis, Funding acquisition, Investigation, Methodology, Project administration, Resources, Software, Supervision, Validation, Visualization, Writing – original draft, Writing – review & editing. KD-O: Data curation, Formal analysis, Investigation, Methodology, Supervision, Writing – review & editing. AZ-G: Data curation, Formal analysis, Methodology, Supervision, Writing – review & editing. AK-K: Data curation, Formal analysis, Methodology, Project administration, Software, Supervision, Validation, Writing – review & editing. KT: Data curation, Formal analysis, Investigation, Methodology, Supervision, Validation, Writing – review & editing. MW: Data curation, Formal analysis, Writing – review & editing, Funding acquisition. DJ: Data curation, Formal analysis, Writing – review & editing. AK: Data curation, Formal analysis, Investigation, Software, Validation, Visualization, Writing – review & editing. AL-A: Data curation, Formal analysis, Investigation, Methodology, Resources, Supervision, Writing – review & editing. EP: Conceptualization, Data curation, Formal analysis, Methodology, Supervision, Validation, Visualization, Writing – review & editing. JW: Data curation, Project administration, Software, Writing – review & editing. JS: Supervision, Writing – review & editing.
